# Use of Micropatterned Thin Film Nitinol in Carotid Stents to Augment Embolic Protection

**DOI:** 10.3390/jfb7040034

**Published:** 2016-12-13

**Authors:** Mahdis Shayan, Brian T. Jankowitz, Puneeth Shridhar, Youngjae Chun

**Affiliations:** 1 Department of Industrial Engineering, University of Pittsburgh, Pittsburgh, PA 15213, USA; mas461@pitt.edu; 2 Department of Neurological Surgery, University of Pittsburgh Medical Center, Pittsburgh, PA 15213, USA; btj1@pitt.edu; 3 Department of Bioengineering, University of Pittsburgh, Pittsburgh, PA 15213, USA; pus8@pitt.edu; 4 McGowan Institute for Regenerative Medicine, Pittsburgh, PA 15213, USA

**Keywords:** thin film nitinol, carotid artery, micro mesh stent, micropatterning

## Abstract

Stenting is an alternative to endarterectomy for the treatment of carotid artery stenosis. However, stenting is associated with a higher risk of procedural stroke secondary to distal thromboembolism. Hybrid stents with a micromesh layer have been proposed to address this complication. We developed a micropatterned thin film nitinol (M-TFN) covered stent designed to prevent thromboembolism during carotid intervention. This innovation may obviate the need or work synergistically with embolic protection devices. The proposed double layered stent is low-profile, thromboresistant, and covered with a M-TFN that can be fabricated with fenestrations of varying geometries and sizes. The M-TFN was created in multiple geometries, dimensions, and porosities by sputter deposition. The efficiency of various M-TFN to capture embolic particles was evaluated in different atherosclerotic carotid stenotic conditions through in vitro tests. The covered stent prevented emboli dislodgement in the range of 70%–96% during 30 min duration tests. In vitro vascular cell growth study results showed that endothelial cell elongation, alignment and growth behaviour silhouettes significantly enhance, specifically on the diamond-shape M-TFN, with the dimensions of 145 µm × 20 µm and a porosity of 32%. Future studies will require in vivo testing. Our results demonstrate that M-TFN has a promising potential for carotid artery stenting.

## 1. Introduction

Approximately 795,000 people suffer from a stroke annually in the U.S. and 15%–20% of these strokes are associated with carotid artery disease [1,2]. These strokes are likely due to a combination of hypoperfusion from stenosis and distal thromboembolism. Carotid endarterectomy (CEA) is the gold standard treatment option for carotid artery stenosis [2]. However, carotid artery stenting (CAS) has become an increasingly popular alternative with recent evidence supporting its equivalence to CEA [3].

CAS consistently shows a higher risk of procedural stroke which is likely due to an increased risk of distal thromboembolism, particularly during angioplasty. Various types of embolic protection devices (EPDs) have been designed to reduce this risk [4]. While the use remains logical and compelling, no randomized studies have been able to prove the efficacy of EPDs for carotid artery stenting [5].

All currently Food and Drug Administration (FDA) approved carotid stents are bare metal, single layer, and have a large pore size. Due to the procedural risk of distal thromboembolism, from atherosclerotic debris extruding through the stent pores, there is currently significant interest in creating a covered stent to trap debris against the blood vessel wall. The cover would optimally need to be porous so it does not occlude side branches such as the external carotid artery. Recently, a CE-marked covered stent, CGuard^TM^ system, consisting of a metallic stent covered with an ultra-thin polyethylene terephthalate (PET) mesh has been studied. A 30-day clinical trial in patients undergoing carotid artery stenting with CGuard^TM^ system demonstrated the safety and efficacy of this device [6]. Another promising stent called Roadsaver^TM^, made up of an ultra-thin nitinol wire micromesh woven onto an external closed-cell stent to achieve a cell size of 375–500 mm, has been recently gaining traction [7]. In both stent frames, the micromesh or double layer technology aims at preventing plaque protrusion [8]. New stent designs incorporating an inner mesh with pore sizes down to below 200 μm may allow additional reduction of lesion-derived microemboli [9]. The current generation carotid stents typically incorporate an open or closed-cell design. Open-cell stents have better conformability and wall apposition but their larger pore size reduces plaque coverage and allows a higher risk of debris extrusion [10]. Recent imaging capabilities, such as Optical Coherence Tomography (OCT) have played an important role in identifying the plaque prolapse [11]. The closed-cell stents have superior scaffolding and plaque coverage due to their smaller pore size, however this increases the probability of mal-apposition, especially within tortuous arteries [12]. A hybrid configuration with an open-cell design at the proximal and distal ends combined with a closed-cell design of the central segments has been recently developed. It not only allows greater apposition to the vessel wall but also offers improved plaque coverage.

In this study, we employ micropatterned thin film nitinol as the covering membrane for a double layer carotid stent. Thin film nitinol (TFN) is a novel material worth considering for developing a low-profile endovascular device [13]. The vacuum sputter deposition process for thin film nitinol is the preferred fabrication method because of the precise controllability of the deposition process and the consistency of the quality of the films [14]. Furthermore, thin film nitinol can be used in small calibre vascular systems as it exhibits superelastic property at body temperature, which improves the device apposition to the vascular wall compared to any synthetic fabric (such as polyethylene terephthalate micronet) that typically has wrinkles or permanent deformation.

In vitro studies were performed to evaluate the feasibility of micropatterned thin film nitinol as a carotid stent cover. We specifically looked at this new stent’s ability to capture embolic particles and inhibit distal embolization of artificial plaque debris. We also studied its interaction with endothelial cells.

## 2. Materials and Methods

### 2.1. Preparation and Characterization of Micropatterned Thin Film Nitinol (M-TFN)

The sputter deposition technique along with a lift-off process previously described by Chun et al. was used to create M-TFN [15]. First, five different micro patterns were defined via a conventional photolithography process. Then, 50 µm deep trenches were created by the Deep Reactive Ion Etching (DRIE) technique. Once trenches were formed, both the copper sacrificial layer and silicon dioxide material inhibition layer were deposited for the thin film nitinol sputter deposition process. The deposited thin film nitinol was crystallized at 500 °C for 20 min in a vacuum of less than 1 × 10^−7^ Torr [16]. Finally, the M-TFN was obtained by etching sacrificial and inhibition layers consecutively. Scanning electron microscopy (SEM) (Jeol-JSM 6610 LV, Tokyo, Japan) was used to characterize the geometry and size of the M-TFN. The obtained SEM images were used to calculate the porosity of the fenestrations in M-TFN with the custom-built MATLAB R2015a (MathWorks, Natick, MA, USA) image analysis code.

### 2.2. In Vitro Testing for Assessing the Efficiency of Embolic Protection Capability

**Figure 1:**
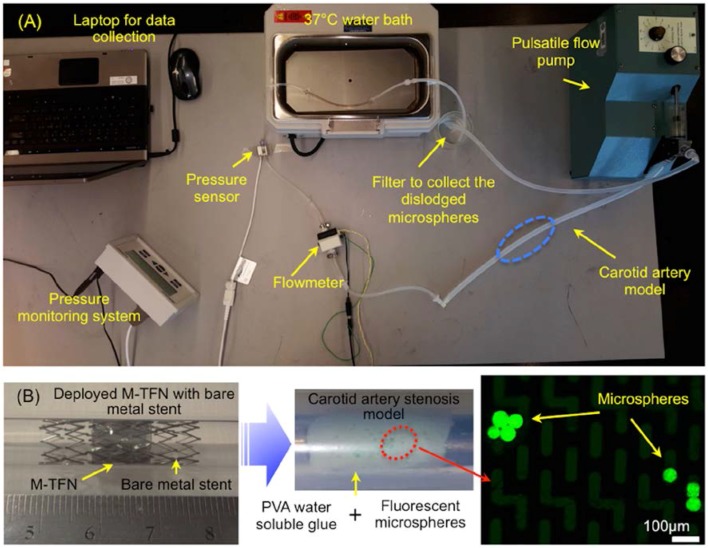
(**A**) In vitro flow loop for assessing the M-TFN covered stent’s performance and (**B**) Deployed M-TFN with a commercially available bare metal stent (**left**); PVA based water-soluble glue layer with fluorescent microspheres (**middle**); and microspheres characterize by microscopy (**right**).

Figure 1A shows a bench-top flow loop equipped with a pulsatile pump used for the in vitro testing for assessing the efficiency of embolic protection capability of the five different M-TFN covering membranes. The capture efficiency relates to the embolic protection efficiency in a sense that the fluorescent microspheres are larger than the pore size of the micropatterned thin film nitinol and the capture efficiencies were obtained under pulsatile flow conditions. This test apparatus consists of platinum cured silicone tubing (Cole-Parmer, Chicago, IL, USA) with an inner diameter of 6.5 mm, which is close to the average human carotid artery dimensions [17]. In order to mimic the carotid artery stenosis, we used gold-standard fluorescent microspheres (Cospheric, Santa Barbara, CA, USA) with different dimensions (e.g., diameter in the range of 53–63 µm) along with the water-soluble polyvinyl alcohol (PVA) glue (Lineco Inc., Holyoke, MA, USA). Microspheres in two different weight loads (i.e., 10 mg and 30 mg) were homogenously dispersed in 500 mg PVA fluid. The PVA fluid that contains microspheres was uniformly deposited on the luminal side of the silicone tubing. The total length of the deposition was determined to be 14 mm based on the longitudinal length of the M-TFN covering membrane used for the experiment. Then, the M-TFN covering membrane was rolled and collapsed into a 6Fr delivery catheter with the commercially available bare metal stent (Pfm Medical, Cologne, Germany). The PFM medical stent was used only for exerting the radial force to the M-TFN under in vitro testing. The stent was deployed on the microsphere mixed PVA fluid covering the strut with the M-TFN membrane, which mimics the embolic protection device of the covered stent. Once the artery models were prepared, these were submerged in the 37 °C water bath, then, connected to the pulsatile flow pump (Harvard apparatus, Holliston, MA, USA). The blood mimicking solution (viscosity of 3.4cP by mixing Distilled water and Glycerol) [18] was applied to achieve realistic circulation of the flow, which circulated at 402 and 510 mL/min and 65 and 85 beats per minute (bpm) in the tube for 30 min [19]. Both flowmeter (FLR1000, Omega, Norwalk, CT, USA) and pressure sensor (PressureMAT, PendoTECH, Princeton, NJ, USA) were integrated adjacent to the carotid artery model to monitor the flow and pressure conditions in real time. At the end of the flow loop, a micromesh filter (Component Supply Co. Inc., Fort Meade, FL, USA) with 105 µm pore dimension (32% opening area) was placed to collect the dislodged microspheres from the wall of the artery model. The microspheres collected by the filter were characterized using a fluorescent microscopy system (Olympus BX43, Olympus Co., Tokyo, Japan) to mimic and quantify the amount of the dislodged emboli under the typical carotid artery ischemic disease conditions. 

### 2.3. In Vitro Cell Culture Experiment

Both non-patterned thin film nitinol (i.e., control sample) and M-TFN were prepared with the dimension of 1 cm × 1 cm. All samples were sterilized by immersion in 70% ethanol for 30 min, then, transferred to a 12-well tissue culture plate (Becton Dickinson and Company, Frankline Lakes, NJ, USA). After transferring the samples to a culture plate, all samples were washed with sterile phosphate-buffered saline (Gibco, Grand Island, NY, USA). BAEC (Bovine Aortic Endothelial Cells; Lonza, Allendale, NJ, USA) at passage number 5 were seeded at a density of 2 × 10^5^ cells/cm^2^ on the samples. Cells were grown in the cell culture medium consisting of Endothelial Cell Basal Medium (EBM)-2 (Lonza, Allendale, NJ, USA) and EGM-2 SingleQuot Kit Suppl. & Growth Factors (Hydrocortisone 0.02%, Fetal Bovine Serum 2%, Vascular Endothelial Growth Factor 0.05%, Human Fibroblast Growth Factor-B 2%, Insulin-like Growth Factor-1 0.05% and Human Epithelial Growth Factor 0.05%, Gentamicin-1000 0.05%) (Lonza, Allendale, NJ, USA). A 2 mL cell culture medium was added in each well of the tissue culture plate and the plates were kept in a humidified incubator under 5% CO_2_ at 37 °C. 

In order to visualize the cell morphology, Alexa Fluor^®^ 488 Phalloidin (Thermo Fisher Scientific, Pittsburgh, PA, USA) was used for the staining of F-actin. After 48 h of cell culture, the samples were washed with PBS, fixed in 3.7% formaldehyde solution in PBS for 10 min at room temperature and then each sample was extracted using acetone at −20 °C for 3–5 min. Subsequently, the samples were loaded with the fluorescent Phalloidin and washed with PBS, and then, images were taken using fluorescent microscopy.

### 2.4. Endothelial Cell Growth Behaviour Study

#### 2.4.1. Quantification of Endothelial Cell Elongation and Alignment

To quantitatively evaluate the cell elongation and orientation, each Phalloidin stained cell was visualized as an elliptical shape. The ratio of the major elliptic axis of the individual stained cells with respect to the horizontal axis was measured using MATLAB R2015a (MathWorks, Natick, MA, USA) image analysis. In addition, the angle between the major axis of each individual cell with respect to the long axis of the micropatterns was measured as the alignment angle using MATLAB image analysis (MathWorks, Natick, MA, USA). Four random locations were characterized for both the five M-TFN samples and control (i.e., nonpatterned film) samples for the analysis (i.e., total 24 images).

#### 2.4.2. MTT (3-(4,5-Dimethylthiazol-2-yl)-2,5-Diphenyltetrazolium Bromide) Endothelial Cell Viability and Proliferation Assay

Cell proliferation was assessed by a colorimetric assay using 3-(4,5-dimethylthiazol-2-yl)-2,5-diphenyl tetrazolium bromide (CT02 MTT Cell Growth Assay Kit: EMD Millipore, Darmstadt, Germany). Cells were cultured for 1 h, 4 h, 24 h and 5 days at an initial seeding density of 2 × 10^5^ cells/mL on both M-TFN and control samples with three replicates of each sample. After determining the incubation time, 0.01 mL pre-warmed MTT solution containing 0.1 mL fresh cell medium was added to each culture well, then, continuously incubated for 4 h under the same conditions. After 4 h incubation of cells with MTT, 0.1 mL isopropanol with 0.04 N hydrochloric acid (HCl) was added to each well. The HCl converts the phenol red in tissue culture medium to a yellow colour that does not interfere with MTT formazan measurement. The isopropanol dissolves the formazan to give a homogeneous blue solution. The absorbance was measured at a wavelength of 570 nm using a microplate reader (BioTek Synergy 2, Winooski, VT, USA). 

#### 2.4.3. Morphology and Coverage Percentage of Endothelial Cells

The morphology of the endothelial cells adhered on each sample was characterized by SEM after a thin layer of palladium deposition by sputtering. For the SEM, cells were fixed with 2.5% glutaraldehyde (Sigma-Aldrich, St. Louis, MO, USA), dehydrated in a series of ethanol/DI water mixtures including 30%, 50%, 75%, 90% and 100% ratios, then, subjected to drying with hexamethyldisilazane (HMDS) (Alfa Aesar, Ward Hill, MA, USA)/ethanol in 3:1, 1:1 and 1:3 volume ratios. The samples were then dried in the hood overnight at room temperature. Coverage percentage of endothelial cells on the M-TFN and the control samples were analysed using MATLAB image analysis based on the SEM images.

### 2.5. Statistical Analysis

The values related to the embolic protection efficiency of the M-TFN, optical density with respect to MTT assay; aspect ratio of attached endothelial cells and endothelial cell coverage percentage were expressed as the mean value ± standard deviation (SD). Statistical analysis was performed for all the above-mentioned experiments using one-way analysis of variance (ANOVA) test and Tukey’s honest significance test (HSD) test by Minitab^®^ 16.1.0 statistical software (Minitab Inc., State College, PA, USA) and a *p*-value < 0.01 was considered to be statistically significant. 

## 3. Results and Discussion

### 3.1. Efficiency of the Embolic Protection Capability (EEPC) of the M-TFN Covered Stents

Table 1 shows the parameters selected for in vitro embolic protection capability study of the M-TFN stents based on the carotid artery stenosis environment described in Section 2.2.

**Figure 2:**
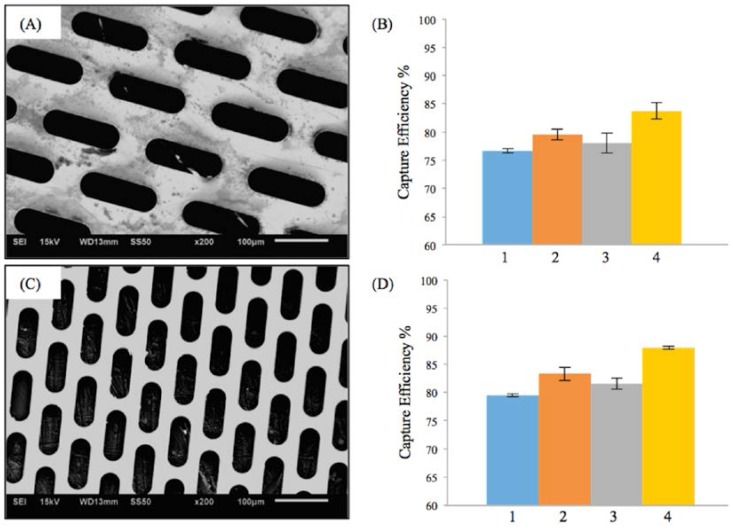
Results on the efficiency of embolic protection with two different sizes of ellipse patterns in thin film nitinol; (**A**) and (**B**) show the fenestration size of 118 μm × 38 μm; (**C**) and (**D**) show the fenestration size of 98 μm × 23 μm.

Figure 2A,C shows the scanning electron microscopy (SEM) images of two different fenestrations with elliptical geometry. The first micropattern (Figure 2A) had a lateral length of 118 μm and vertical length of 38 μm. The porosity of first M-TFN sample was 44%. The second ellipse (E) micropattern had a lateral length of 98 μm and vertical length of 23 μm, showing 39% of porosity. While both E-patterns have different pore size and porosity, the embolic protection efficiencies were found to be very similar.

Figure 2B,D represents the efficiency of the embolic protection capability, i.e., the percentage of the number of particles isolated by the M-TFN covering membrane compared with the total number of particles. The particles left on the wall of carotid artery model by the first pattern of M-TFN were seen to decrease with the higher flow rates (i.e., conditions 1 and 3 in Figure 2B). The higher flow rates in the same diameter artery model resulted in an increase in the flow velocity as well as shear rate on the wall of the model. Therefore, condition 1 had less number of particles left on the wall showing almost similar EEPC of 76.65% and 79.48%, respectively. Condition 3 also showed EEPC of 78.02% and 81.52% in each sample, respectively. The highest EEPC among both E-patterned samples was found in condition 4 showing 83.74% and 87.94%, respectively.

**Figure 3:**
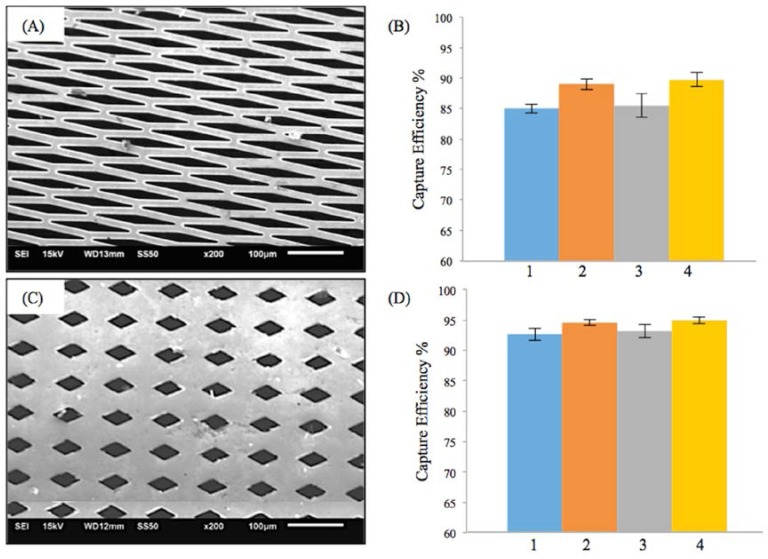
Results on the efficiency of embolic protection with two different sizes of diamond patterns in thin film nitinol; (**A**) and (**B**) show the fenestration size of 145 μm × 20 μm; (**C**) and (**D**) show the fenestration size of 55 μm × 30 μm.

Similar trends as in M-TFN with E-patterns were found in M-TFN with diamond shaped (D) micro patterns. Figure 3A,C shows two different types of D-patterns created in TFN. The first pattern (Figure 3A) has 32% porosity with a lateral length of 145 μm and vertical length of 20 μm. The second pattern (Figure 3B) has 17% porosity with a lateral length of 55 μm and vertical length of 30 μm. Similar to EEPC seen in E-patterns, higher flow rates resulted in the decrease in EEPC for the first D-pattern (Figure 3B). However, the EEPCs were very similar for the D-patterned TFN with 17% of porosity. Compared with the E-patterns, D-patterns showed better EEPC with slightly decreased porosity. For example, while the second E-pattern (Figure 2C) with 39% porosity showed EEPC of 79.49%, 83.30%, 81.52%, and 87.94% for the conditions from 1 to 4, respectively, the first D-pattern (Figure 3A) with 32% porosity showed EEPCs of 85.01%, 89.02%, 85.48%, and 89.77%. Also, it was noted that M-TFN, with much less porosity, showed higher EEPCs indicating 92.67%, 94.59%, 93.25%, and 94.95% for the conditions from 1 to 4, respectively. 

**Figure 4:**
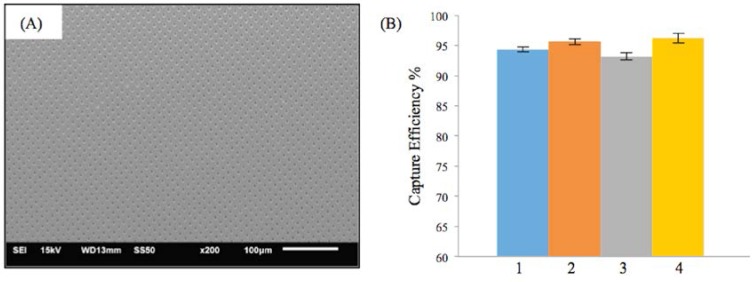
Results on the efficiency of embolic protection with circular pattern in thin film nitinol; (**A**) and (**B**) show the fenestration size of 5 μm in diameter.

Figure 4A shows the SEM image of the M-TFN with circular (C) patterns with the diameter of 5 μm. The porosity of the C-pattern was 15%, which was slightly less than the porosity found in the low porosity D-patterns (Figure 3C). The EEPCs showed a very similar trend, as observed in D-patterns (Figure 3D). The C-pattern showed EEPCs 94.36%, 95.61%, 93.15%, and 96.18% for the conditions from 1 to 4, respectively. There was no significant difference between the two samples, i.e., Figure 3D and Figure 4B, representing the low porosity below ~17% which does not actually represent an increase in the EEPC. In addition, we may note that the M-TFNs with lower porosity (Figure 3C and Figure 4A) exhibit longitudinal stiffness, as well as such patterns being virtually impossible to collapse into a small delivery catheter. Therefore, the optimal micro pattern will be the D-pattern with flexible TFN, which is represented in Figure 3A.

### 3.2. Biocompatibility Assessment via In Vitro Endothelial Cell Growth Behaviour

Biocompatibility of M-TFN as a covering membrane for carotid stent should be evaluated to demonstrate the efficacy of the material in use. In this study, endothelial cell growth behaviour studies were conducted to assess (1) cell viability and proliferation with TFN using MTT assay; (2) the shape of the grown cells on TFN using both fluorescent microscope and scanning electron microscope image analyses; (3) the orientation of cells grown on TFN; and (4) the morphologies of the grown cells on TFN. The D-pattern in Figure 3A was chosen for endothelial cell growth behaviour studies because this pattern showed appropriate EEPC as well as flexible device design capabilities.

**Figure 5:**
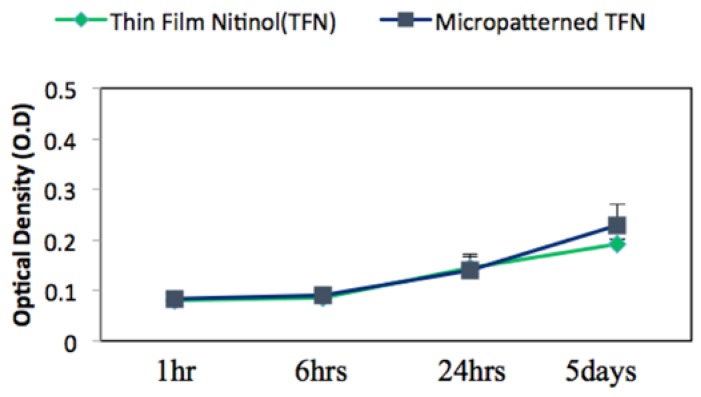
MTT assay for endothelial cell viability cultured on the TFN and micropatterned TFN substrates after 1 h, 2 h, 4 h, 24 h and 5 days.

Figure 5 represents the mean value of endothelial cell’s viability on TFN and M-TFN samples that were evaluated using the MTT assay over five different time periods (1 h, 2 h, 4 h, 1 day and 5 days). After 1 h, 2 h, 4 h and 1 day, no significant difference in optical density was observed between the TFN and M-TFN samples. After 5 days, however, the optical density was seen to be on the rise on the M-TFN sample (value = 0.2273). It was 18.38% higher compared with the density found in the nonpatterned TFN (value = 0.192). Overall, our short-term in vitro tests showed better endothelial cell proliferation performance in M-TFN rather than non-patterned TFN (*p*-value < 0.01). 

### 3.3. Quantification of the Endothelial Cell’s Growth Pattern and Orientation

**Figure 6:**
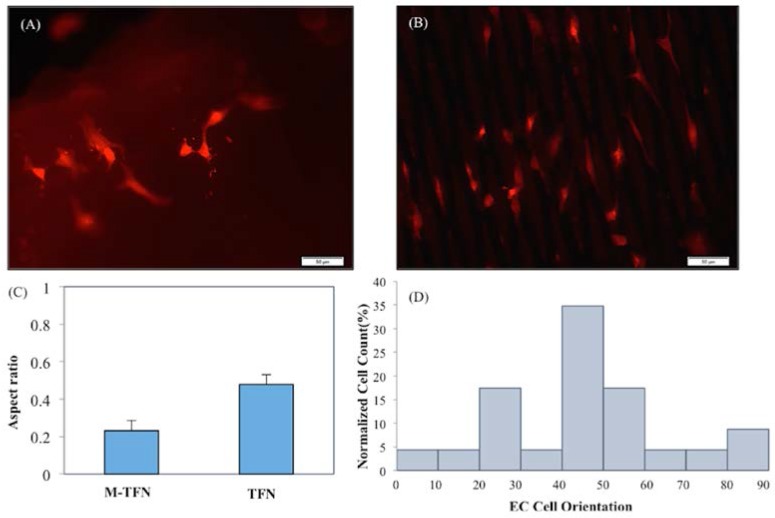
Representative Fluorescent images of F-actin stained endothelial cell cultured on (**A**) TFN; (**B**) M-TFN after 48 h; (**C**) aspect ratio of the grown endothelial cells on TFN and M-TFN; and (**D**) orientation angles of the endothelial cells grown on TFN and M-TFN after 48 h.

Figure 6 shows the representative fluorescent images of stained endothelial cells cultured on (A) TFN and (B) M-TFN after 48 h. As illustrated in Figure 5, MTT assay showed more endothelial cells adhered on the M-TFN along the line of the micro patterns in TFN. The scale bars are depicted in 50 μm. Figure 6C shows the aspect ratio (i.e., ratio of the minor axis to the major axis) of the cells found on the films. The aspect ratio of 1 would mean a symmetric geometry in *x*-axis and *y*-axis. Therefore, as illustrated in Figure 6C, M-TFN showed longer shape of cells with 0.232 aspect ratio compared with non patterned TFN (0.479), indicating that endothelial cells have a tendency to grow along the line of TFN.

Figure 6D shows the distribution of alignment angles of endothelial cells along the major axis of M-TFN. Quantitative analysis of actin alignment of endothelial cells on the M-TFN showed that approximately 35% of cells were oriented along the major axis of the micro patterns with the angle of 40°–50°. Almost 17% of cells were grown with 20°–30° and 17% with 50°–60° orientations. This finding shows that the endothelial cells on M-TFN were grown along the line of the TFN area supporting the observations derived from Figure 6C.

### 3.4. Morphology and Coverage Percentage of Endothelial Cells

**Figure 7:**
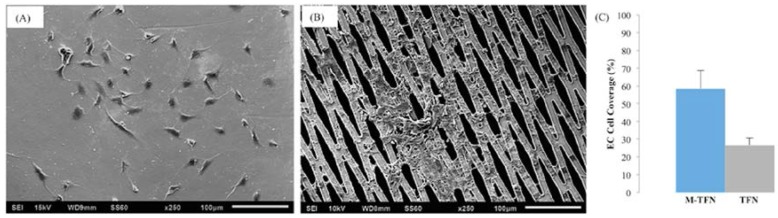
Representative SEM images of endothelial cells cultures on (**A**) TFN and (**B**) M-TFN; and (**C**) percentage of endothelial cells covered on M-TFN and TFN samples.

Figure 7 shows the representative SEM images of endothelial cells cultured on both TFN and M-TFN. Endothelial cells are seen spread out on the TFN substrate and major podia have emerged on the surface of cells (Figure 7A). For M-TFN samples, as in Figure 7B, a few cells were observed on the TFN area and more aggregated endothelial cells were found in the middle, demonstrating that porous regions are also covered with cells over time. The number of grown cells was counted with the SEM images. Figure 7C shows a higher number (120%) of grown endothelial cells in M-TFN compared with TFN. In general, the M-TFN exhibits higher surface-to-volume ratio, therefore, there is an increased tendency for cells to adhere on this sample. In addition, micro patterns actually enhance the cell growth behaviour by providing complex structure where cells are more easily attached.

Covered stents are an attractive way to treat carotid artery stenosis. Following promising results from small case series, a prospective multicentre European trial (Clear Road Trial) showed that the multi-layered Roadsaver^TM^ stent is safe and effective in high risk subjects for carotid endarterectomy [20,21]. Another multi-centre CAS study with 150 patients showed a lower rate of plaque prolapse and favourable short-term outcome [22]. The increased pore density of “mesh stents” promises even higher plaque containment by either avoiding, or at least limiting, plaque prolapse through the cell struts [23].

Thin film nitinol is a promising candidate for use in endovascular devices [24]. Its super low profile minimizes interruption of the vascular lumen and would not disturb the blood flow. It maintains superior versatilibility compared to micromesh technology. Kedev et al. suggested that it is possible that the unique double-layer micromesh design of these stents may obviate the need for systematic embolic protection [25]. However, recent clinical studies such as Confidence Trial have been designed to include both dual layer stents and embolic protection devices (e.g., Nanoparasol).

Covered the stent to prevent embolization in carotid artery stenosis would be ideal because the stent can physically isolate the embolic particles without any additional filter devices or complex steps of procedures. However, a covered stent is not flexible enough to be delivered in some tortuous locations; they also add bulkiness, which may possibly complicate its delivery in an endovascular procedure. In addition, fenestrations should be present in the overlying covering membrane to allow nutrition and oxygen transport from the blood stream to the cells on the wall of the vascular system.

In this study, we introduced a novel covering membrane material, i.e., thin film nitinol. The thin film nitinol has 6μm thickness, and thus could be an ideal candidate for use in low-profile devices for carotid artery stenosis. In addition, various shapes and sizes of pores can be created in the thin metallic film using a lift-off process. As a carotid embolic protection stent, we have shown the relationship among the efficiency of embolic protection, the geometry, and size of microsized patterns created in thin film nitinol. We have demonstrated that the thin film nitinol covering membrane, patterned in various morphologies (i.e., ellipse, diamond and circle) with different porosities (i.e., 44%, 39%, 32%, 17%, and 15%) can isolate embolic particles efficiently (~>75%) during a 30 min in vitro flow test. In our tests, lower flow rates resulted in the increase in capture efficiency or efficiency of embolic protection capability. Furthermore, lower porosity was conducive to improve the efficiency of embolic protection capability. We believe that there is a lower limit for porosity below which no improvement in EEPC may be seen. Among circular (C), diamond (D), elliptical (E) shaped micro-patterns, the diamond shape with a lateral length of 145 μm and vertical length of 20 μm (D-pattern in Figure 3A) is the most favourable in terms of embolic protection efficiency. Also, smaller micro patterns increase the stiffness of the collapsed stent, or even to the extent of not being collapsible into a small delivery catheter.

One other important design criteria for the success of the carotid artery stent is the reduction of the risk of thrombosis and restenosis occurrence which are two major complications leading to the failure of the endovascular devices [26,27,28,29,30]. During a restenosis event, arterial lumen is narrowed again due to the migration of smooth muscle cells and thickening of the scar tissue. Growing a layer of healthy endothelial cells on the thin film nitinol cover and stent struts not only develops a normal lining allowing the normal blood flow, but it also reduces the probability of thrombosis or neointimal hyperplasia. This is achieved by formation of a native vascular endothelium that protects the vessel wall from smooth muscle cell migration and proliferation as well as inflammatory cell infiltration [31].

A number of studies have shown that endothelial cellular response (e.g., adhesion, proliferation) favourably changes on the micro-patterned substrate [32,33]. It is now well-known that micropatterned substrates can significantly modulate cellular responses [34,35,36]. In the case of the smooth surface of a biomaterial substrate (e.g., TFN), cells encounter a homogenous substrate which does not have many important features of the native tissue microenvironment. In the native tissues, each cell is normally surrounded by neighbouring cells within an extracellular matrix (ECM) that constitutes the 3D microenvironment, imposing both geometrical and topographical constraints. The ECM ligands binding with the cell surface receptors not only affect the intracellular signalling pathways, they can also impact the cytoskeleton architecture, e.g., actin network, cell polarity, migration, differentiation and growth.

In our study, in vitro results demonstrated that more than twice the endothelial cell coverage can be seen on micropatterned thin film nitinol compared to the nonpatterned thin film nitinol surface. Also, our short-term endothelial cell proliferation test results point in the direction of M-TFN when compared to non-patterned TFN. The alignment and orientation is related to cytoskeleton organization, e.g., actin aligning and it plays a very important role in the cellular function, such as cell proliferation [37,38]. The majority of the endothelial cells are aligned at an angle of 40°–50° along the axis of the micropatterns. In theory, M-TFNs have higher surface-to-volume-ratios compared to nonpatterned TFNs. We have demonstrated that this holds true in our case. This property is particularly useful and ideal to enhance cell growth behaviour in micromesh carotid stents. However, more studies are needed to find out the longer-term relationship between the endothelial cell shape and its functional behaviour, as well as in vivo tests. Other commercially available stents, such as Acculink (Abbott Laboratories, Abbott Park, IL, USA), Precise (Cordis Corporation, Miami, FL, USA), or Xact (Abbott Laboratories, Abbott Park, IL, USA) stents should be used for future in vivo animal testing (not in this study). One of the major limitations of our work is that we utilized fluorescent microspheres instead of human plaque. In addition, the plaque specimens or fluorescent microspheres of smaller sizes (especially less than 50 µm) were not filtered. The impact of very small emboli needs to be understood and remains unknown.

## 4. Conclusions

We have shown that micropatterned thin film nitinol based covered stents are promising biomaterials for the fabrication of dimensionally and geometrically well-defined, carotid artery stents. The effective geometric properties (especially diamond shaped patterns) and the porosities of the micropatterned thin film substrate can be extremely important to improve the efficiency of the embolic prevention. This capability, in turn, improves the stroke prevention profile of the carotid stent and obviates the requirement of embolic protection devices. The micropatterned TFNs are highly biocompatible. The elongation, alignment, viability, and proliferation of the endothelial cells, along with the morphology and coverage, are controlled by the presence of micropatterns in the TFN substrate. All these properties together highlight the suitability of the micropatterned thin film nitinol for hybrid carotid stents for endovascular applications, where the material must be able to prevent both early and late embolization without blocking side arteries and exhibit high flexibility to ensure easy deployment for the utility during an intervention. In vivo studies must be carried out to demonstrate whether these preliminary results might translate into relevant clinical benefits as compared to the current available technologies.

## Figures and Tables

**Table 1:** Parameters used for assessing the efficiency of embolic protection capability (EEPC).

Test Condition	Flow Rate (mL/min)	Pulsatile Rate (Beats per min)	Weight of Microspheres (mg)	PVA Solution (mg)	Particle Size (μm)
1	510	85	30	500	53–63
2	402	65	30	500	53–63
3	510	85	10	500	53–63
4	402	65	10	500	53–63
